# Selective Laser Spectroscopy of the Bixbyite-Type Yttrium Scandate Doped by Rare-Earth Ions

**DOI:** 10.3390/ma16216829

**Published:** 2023-10-24

**Authors:** Elena Dobretsova, Olimkhon Alimov, Sergey Rusanov, Vitaly Kashin, Vladimir Tsvetkov

**Affiliations:** Prokhorov General Physics Institute of the Russian Academy of Sciences, 119991 Moscow, Russia; olim@lst.gpi.ru (O.A.); rusanov@lsk.gpi.ru (S.R.); kashin@lsk.gpi.ru (V.K.); tsvetkov@lsk.gpi.ru (V.T.)

**Keywords:** selective laser spectroscopy, optical center, rare-earth ion

## Abstract

Yttrium scandate crystals doped by Nd^3+^ and Tm^3+^ ions have been successfully grown in the form of fibers using the laser-heated pedestal growth (LHPG) technique. The selective laser spectroscopy methods have identified and distinguished three distinct types of optically active centers associated with Nd^3+^ and Tm^3+^ ions. The substitution of Y^3+^ and Sc^3+^ for rare-earth ions in the *C*_2_ structural site leads to the formation of two distinct basic long-time centers. In Nd^3+^:YScO_3_, another type of center (a short-lifetime one) is formed known as the Nd^3+^–Nd^3+^ aggregate pair. This center arises from the substitution of Y^3+^ or Sc^3+^ for Nd^3+^ cation in the adjacent *M*O_6_ polyhedra that share an edge. In Tm^3+^:YScO_3_, the third optical center is formed as a result of the substitution of Y^3+^ or Sc^3+^ for Tm^3+^ in the *M*O_6_ octahedra with the *C*_3*i*_ site symmetry. The fluorescence decay lifetimes of Nd^3+^ and Tm^3+^ ions in the YScO_3_ crystal structure have been accurately measured and estimated. A Stark level diagram illustrating the splitting of ^4^*F*_3/2_, ^4^*I*_11/2_, and ^4^*I*_9/2_ multiplets of Nd^3+^ ions has been constructed to show features of the active optical centers with the *C*_2_ site symmetry.

## 1. Introduction

Rare-earth (*RE*^3+^)-doped Sc_2_O_3_ and Y_2_O_3_ sesquioxides exhibit high thermal conductivity, approximately twice that of yttrium aluminum (YAG) [1], and have a wide transparency range from 0.25 μm to 9.6 μm [2]. These materials have demonstrated excellent potential for high-power solid-state lasers [3,4,5,6,7,8], particularly in the infrared (IR) spectral region. They could serve as a viable alternative to the well-established YAG crystals. *RE*^3+^-doped sesquioxide materials show strong potential for a variety of applications, including solid-state light-emitting devices [9,10,11], high-efficiency luminescent materials [12,13], rare-earth magnets [14,15], magneto-optical recording materials [16,17], and so on.

Both Y_2_O_3_ and Sc_2_O_3_ are categorized as a cubic bixbyite-type structure [18,19,20,21,22], where there are one position for the oxygen ion and two symmetrically independent positions for cations: the 8*b* site with *C*_3*i*_ symmetry and 24*d* site with *C*_2_ symmetry. The unit cell contains three cations occupying the the *C*_2_ site and one cation in the *C*_3*i*_ site. Two types of 6-vertex polyhedra share corners and edges to form a chessboard packing derived from the fluorite structure type [23].

Extensive research has been carried out on rare-earth dopants in Y_2_O_3_ crystals, revealing that *RE*^3+^ ions have the capability to occupy Y^3+^ sites [24,25,26,27,28,29,30,31]. However, due to inversion symmetry, the electric dipole–dipole transitions within the 4*f*-configuration are forbidden for the Kramers *RE*^3+^ ions (which have an odd number of 4*f*-electrons) occupying the *C*_3*i*_ sites. Consequently, all electron transitions observed in the fluorescence spectra of these ions should be assigned to electronic transitions in active optical centers located in the *C*_2_ sites. Sc_2_O_3_ is also a promising host material as it exhibits a strong crystal field effect, primarily caused by the lattice distortions resulting from the substitution of Sc^3+^ for *RE*^3+^ ions of larger size [4,32,33]. Moreover, Tm^3+^-doped Sc_2_O_3_ and Y_2_O_3_ have extraordinary broad gain spectra around 2 μm. In comparison to YAG, the peak emission cross-section of the Tm^3+^ ion at 2 μm is four times greater in both Sc_2_O_3_ and Y_2_O_3_ [32].

Yttrium scandate (YScO_3_) has a polymorphic nature, implying that the laser properties can be varied depending on the host crystal structure and the type of rare-earth ions. Additionally, mixed sesquioxides demonstrate notably lower melting temperatures compared to yttria and scandia. Mixed sesquioxides hold great promise for the generation and amplification of ultrashort pulses [6,34,35]. This is due to their inhomogeneously broadened gain spectra, which is a result of the intrinsic disorder in the crystal structure [35]. Nd lasers have been realized successfully in the YScO_3_ and isostructural (Lu_1−x_Sc_x_)_2_O_3_ mixed seqioxides [36]. The high-temperature modification of YscO_3_ belongs to the bixbyite-type structure [18,20,37,38] similar to yttria (Y_2_O_3_) [21] and scandia (Sc_2_O_3_) [22]. It can be “frozen” during the crystal growth process, and the synthesis method used plays a critical role. The features of the laser-heated pedestal growth (LHPG) technique are high temperature of heating under laser irradiation followed by rapid cooling. The method allows one to obtain crystals in the form of fibers [39,40].

There is a high probability that certain rare-earth optical centers will be formed in the disordered structure. Selective laser spectroscopy methods are particularly suitable for studying optical properties of rare-earth-doped laser active media such as yttrium scandate.

We have conducted a comprehensive spectral–kinetic analysis of both Nd^3+^:YScO_3_ and Tm^3+^:YScO_3_ crystal fibers using the methods of selective laser spectroscopy.

## 2. Materials and Methods

Crystals of 0.1 at. % Nd^3+^:YScO_3_ and 0.1 at. % Tm^3+^:YScO_3_ have been obtained using the laser-heated pedestal growth (LHPG) technique. Commercial powders of Y_2_O_3_ and Sc_2_O_3_ (purity > 99.999%, Sigma Aldrich, Burlington, MA, USA) were used as precursors, while Nd_2_O_3_ and Tm_2_O_3_ powders (purity > 99.999%, Sigma Aldrich, Burlington, MA, USA) were used as activators. Both Nd^3+^:YScO_3_ and Tm^3+^:YScO_3_ crystals have been grown in the form of fibers with a diameter of 0.6 mm and a length of 50 mm.

The structural characteristics of the Nd^3+^- and Tm^3+^-doped YScO_3_ crystal fibers have been described in our earlier works [18,37,38]. The X-ray pattern of YScO_3_ includes good narrow peaks, which points to a high degree of crystallinity. According to X-ray data, the YScO_3_ crystal fiber is homogeneous and belongs to the bixbyite structural type (space group *Ia*3).

Time-resolved luminescence and excitation spectra and kinetics of luminescence decay of both Nd^3+^ and Tm^3+^ ions in YScO_3_ crystal fiber were measured on the MDR-23 monochromator (LOMO, Saint-Petersburg, Russia) at temperatures of 77 and 300 K. The Solar LP-604 parametric generator (Solar LS, Minsk, Belarus) was used as a source of excitation. The luminescence excitation spectra were measured by scanning the irradiation of the Solar LP-604 tunable parametric generator across the absorption bands of both Nd^3+^ and Tm^3+^ ions while keeping the absorption intensity of the laser emission on the studied transitions constant (*λ*_det_. = const). The decay kinetics of luminescence was measured using the double selection method (based on luminescence spectrum and excitation). The luminescence from the studied samples was focused into the slit of the monochromator using a condenser, and the signal was subsequently detected using the FEU-83 and Hamamatsu R5108 photomultipliers (Hamamatsu Photonics, Sendai City, Japan) and a PbS photosensitive resistor. The signal from the detectors was fed into the Tektronix–TDS3052B wideband (0–500 MHz) oscilloscope (Tektronix, Beaverton, OR, USA) connected to a computer.

All measurements were carried out at temperatures of 77 and 300 K. The width of the spectral lines depends on the value of an electron—the phono interaction of the impurity center with the phonon field of the crystal lattice. In spectroscopy, this parameter is characterized by homogeneous broadening. At 77 K, phonons are frozen up to the energy of kT = 51 cm^−1^, resulting in the narrowing of luminescence lines and a decrease in the value of homogeneous linewidth. At 300 K, phonons with energy up to kT = 210 cm^−1^ are involved, resulting in an increase in nonradiative relaxation channels, leading to broadening of luminescence lines. This effect is especially pronounced for lines in the long-wavelength region of the luminescence spectrum where inter-Stark transitions of both Nd^3+^ and Tm^3+^ ion levels are involved in nonradiative relaxation processes.

## 3. Results and Discussion

### 3.1. Spectroscopy of Nd^3+^:YScO_3_

**Figure 1:**
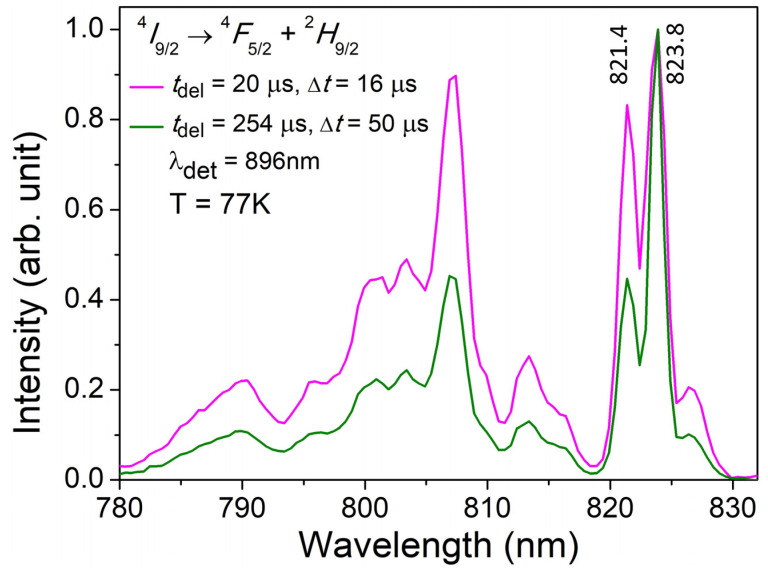
Low-temperature (77 K) time-resolved luminescence excitation spectra of Nd^3+^ ion in YScO_3_ host being measured in the ^4^*I*_9/2_ → ^4^*F*_5/2_ + ^2^*H*_9/2_ electron transitions. A delay time and a gate used were *t*_del(1)_ = 20 μs and Δ*t*_(1)_ = 16 μs, respectively; *t*_del(2)_ = 254 μs and Δ*t*_(2)_ = 50 μs, respectively.

Normalized time-resolved luminescence excitation spectra of Nd^3+^ ions in the YScO_3_ host were recorded at a temperature of 77 K, as shown in Figure 1. The spectra have been measured in the spectral range of 780–850 nm. Luminescence excitation spectra have been obtained through the scanning of parametric laser radiation on the ^4^*I*_9/2_ → ^4^*F*_5/2_ + ^2^*H*_9/2_ electron transitions and selective detection of luminescence on the ^4^*F*_3/2_ → ^4^*I*_9/2_ (*λ*_det_. = 896 nm). The luminescence excitation spectra are modified and intensities between spectral lines, in particular 821.4 and 823.8 nm, are redistributed with changes in delay times of 20 μs to 254 μs relative to the moment of laser excitation. Analysis of the changes in luminescence excitation spectra allows us to assume that two types of Nd^3+^ optical centers exist in the YScO_3_ host.

**Figure 2:**
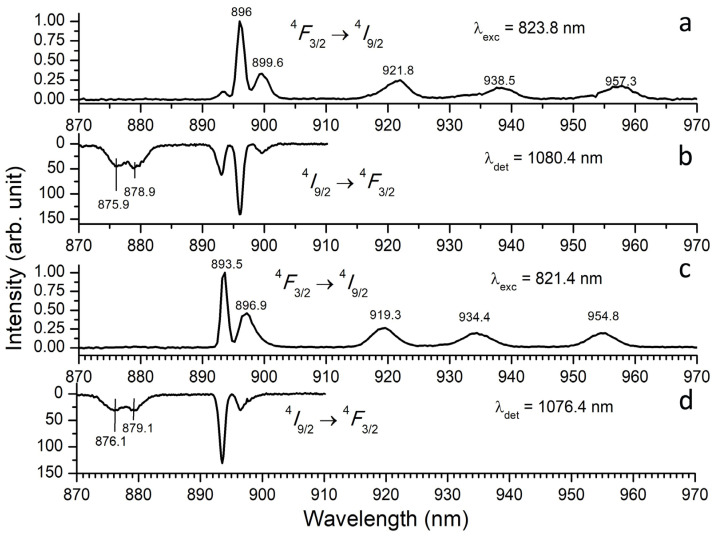
(**a**,**c**) Low-temperature time-resolved luminescence spectra of Nd^3+^ ions in YScO_3_ being measured on the ^4^*F*_3/2_ → ^4^*I*_9/2_ electron transition at excitation of 823.8 nm (center I) and 821.4 nm (center II), respectively. (**b**,**d**) Luminescence excitation spectra of Nd^3+^ ions being measured on the ^4^*I*_9/2_ → ^4^*F*_5/2_ + ^2^*H*_9/2_ electron transitions at detection on 1080.4 nm (center I) and 1076.4 nm (center II), respectively. For all spectra, a delay time and a gate used were *t*_del_ = 10 μs and Δ*t* = 6 μs, respectively.

Time-resolved luminescence spectra of Nd^3+^ ion in the YScO_3_ crystal fiber have been measured on the ^4^*F*_3/2_ → ^4^*I*_9/2,11/2_ electron transitions at 77 and 300 K. Nd^3+^ ions have been excited at 823.8 and 821.4 nm on the ^4^*I*_9/2_ → ^4^*F*_5/2_ + ^2^*H*_9/2_ electron transitions. Figure 2a,c show luminescence spectra of Nd^3+^ on the ^4^*F*_3/2_ → ^4^*I*_9/2_ electron transition being measured at excitation of 823.8 and 821.4 nm, respectively. Luminescence spectra of Nd^3+^ ions change with the excitation wavelength. Double selection for excitation wavelength and time leads to shifting luminescence lines. Double selection shows that luminescence spectra of Nd^3+^ ions change with the excitation wavelength and delay times relative to the moment of laser excitation. Analysis of luminescence spectra of Nd^3+^ ions allows us to determine the number of spectral lines being recorded at two different excitation wavelengths. In both cases, the spectra of Nd^3+^ on the ^4^*F*_3/2_ → ^4^*I*_9/2_ electron transition includes five Stark components (T = 77 K) that correspond to the number of lines (2*J* + 1)/2 being predicted by theory. This points to complete removal of degeneration of the electron transitions.

So, the ^4^*I*_9/2_ electron level splits into five components. ^4^*F*_3/2_ and ^4^*I*_11/2_ split into two and six Stark components, respectively, at each excitation wavelength. Spectral lines are shifting with excitation wavelength, but its number remains the same. This fact points to the presence of two types of Nd^3+^ optical centers. The number of Stark components and values of splitting allow us to conclude that both centers have low site symmetry of *C*_2_.

**Figure 3:**
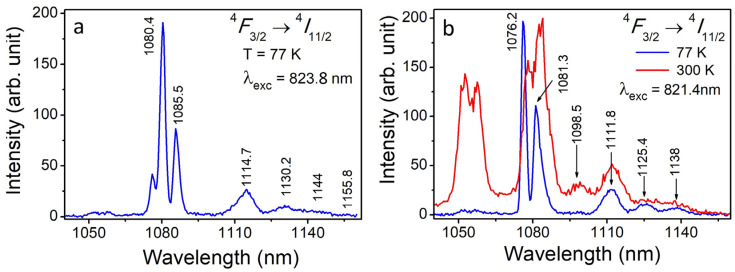
(**a**) Luminescence spectrum of Nd^3+^ ions in YScO_3_ crystal fiber being measured on the ^4^*F*_3/2_ → ^4^*I*_11/2_ at an excitation of 823.8 nm (^4^*I*_9/2_ → ^4^*F*_5/2_ + ^2^*H*_9/2_ electron transition, center I), a delay time of 10 μs, a gate of 6 μs, and a temperature of 77 K. (**b**) Luminescence spectra of Nd^3+^ ions in YScO_3_ crystal fiber being measured on the ^4^*F*_3/2_ → ^4^*I*_11/2_ at excitation of 821.4 nm (^4^*I*_9/2_ → ^4^*F*_5/2_ + ^2^*H*_9/2_ electron transition, center II), a delay time of 10 μs, a gate of 6 μs, and a temperature of 77 and 300 K.

Normalized luminescence spectra of Nd^3+^ ions in YScO_3_ have been measured on the ^3^*F*_3/2_ → ^4^*I*_11/2_ electron transition at excitation of 823.8 and 821.4 nm and temperatures of 77 and 300 K (Figure 3a,b). Analysis of luminescence spectra of the ^3^*F*_3/2_ → ^4^*I*_11/2_ shows that the increase in time delay relative to the moment of laser excitation leads to a shift of spectral lines and redistribution of their intensities on the ^3^*F*_3/2_ → ^4^*I*_9/2_ electron transition. The changes in luminescence spectra allow us to identify spectral lines of Nd^3+^ ions and distinguish two group of lines and hence two types of Nd^3+^ optical centers in the YScO_3_ host.

**Figure 4:**
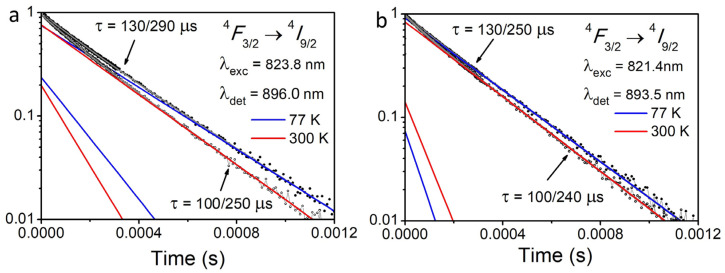
(**a**) Luminescence quenching kinetics of Nd ^3+^:YSO_3_ being recorded at excitation of 823.8 nm (^4^*I*_9/2_ → ^4^*F*_5/2_ + ^2^*H*_9/2_), detection wavelength of 896.0 nm (^4^*F*_3/2_ *→* ^4^*I*_11/2_ electron transition), and temperatures of 77 and 300 K. (**b**) Luminescence quenching kinetics of Nd ^3+^:YSO_3_ being recorded at excitation of 821.4 nm (^4^*I*_9/2_ → ^4^*F*_5/2_ + ^2^*H*_9/2_), detection wavelength of 893.5 nm (^4^*F*_3/2_ *→* ^4^*I*_11/2_ electron transition), and temperatures of 77 and 300 K.

Figure 4a,b show luminescence decay kinetics of Nd^3+^:YSO_3_ being measured at excitation of 823.8 and 821.4 nm (^4^*F*_9/2_ → ^4^*F*_5/2_ + ^2^*H*_9/2_ electron transition) and temperatures of 77 and 300 K. The LP-604 (Solar LS) parametric generator has been used as an excitation source. The luminescence kinetics have been recorded on the ^4^*F*_3/2_ → ^4^*I*_9/2_ electron transition. Approximation of luminescence decay curves of Nd^3+^:YScO_3_ being measured at excitation of *λ*_exc_ = 823.8 nm, detection of λ_det_ = 896.0 nm, and temperatures of 77 and 300 K shows that each of the decay curves is described by two exponential models, with lifetimes of τ_1_(300 K) = 100/250 μs; τ_1_(77 K) = 130/290 μs (Figure 4a). At excitation of *λ*_exc_ = 821.4 nm and detection of *λ*_det_ = 893.5 nm, luminescence decay curves consist of two exponents with lifetimes of τ_2_(300 K) = 100/240 μs and τ_2_(77 K) = 130/250 μs (Figure 4b).

Total intensity of luminescence decay kinetics of Nd^3+^:YScO_3_ is determined by the long-lifetime center (intensity ratio *I*_1_/*I*_2_ of 0.78/0.22, Figure 4a). The short-lifetime center is formed less effectively and its contribution to the total intensity is insignificant (intensity ratio *I*_1_/*I*_2_ of 0.94/0.06, Figure 4b). Figure 4a,b show that the lifetime of both centers is increasing with decreasing temperature down to 77 K. Decreasing temperature probably leads to decreasing electron–phonon interaction and intracenter nonradiative relaxation of the excitation energy.

The crystal structure of Nd^3+^:YScO_3_ has been described previously [18]. It was shown that neodymium ions can substitute basic ions of yttrium and scandium. The relation between ionic radii of yttrium (0.9 Å), scandium (0.83 Å), and neodymium (0.98 Å) is near the Goldschmidt criterion. Substitution of yttrium and scandium ions by neodymium does not require charge compensation in YScO_3_. This leads to the formation of two basic types of optical center of Nd^3+^ activators with the local symmetry of *C*_2_. However, a slight difference between cation radii leads to a local distortion in octahedra occupied by Nd^3+^. The selective laser spectroscopy method is sensitive to these changes, and local distortion in the ion activator surrounding affects the spectral–kinetic characteristics of neodymium ion.

The third optical center with a short lifetime is probably the pair aggregate (Nd^3+^–Nd^3+^) one. The Nd^3+^–Nd^3+^ center is formed in the neighboring *M*O_6_ polyhedra sharing the O^2−^–O^2−^ edge, where Nd^3+^ substitutes basic ions Y^3+^ and Sc^3+^. The multipole interaction of Nd^3+^ ions in the pair (Nd^3+^–Nd^3+^) leads to luminescence quenching and hence decreasing lifetime of the Nd^3+^ activator. The effect of this interaction depends on activator concentration as well as Nd^3+^–Nd^3+^ distance. This is one of the reasons for the non-exponential character of kinetics at the beginning state of the luminescence quenching.

Spectral lines of the Nd^3+^ ion occupying the *C*_3i_ site are not observed because electro-dipole transitions are forbidden in rare-earth ions occupying centro-symmetric positions.

**Figure 5:**
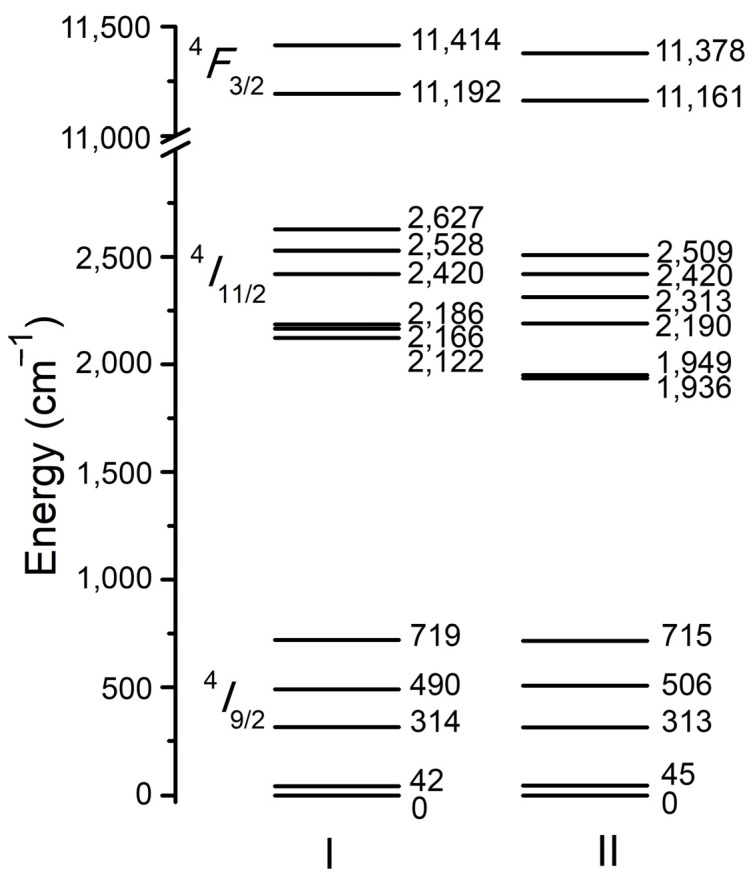
Scheme of ^4^*I*_9/2_,^4^*I*_11/2_, and ^4^*F*_3/2_ Stark levels for two types of Nd^3+^ centers with a site symmetry of *C*_2_ (I) and *C*_2_ (II) in YScO_3_ crystal structure.

Analysis of luminescence excitation (^4^*I* _9/2_ → ^4^*F*_5/2_ + ^2^*H*_9/2_ transitions) and luminescence spectra (^4^*F*_3/2_ → ^4^*I*_9/2_, ^4^*I*_11/2_ transitions) allows us to build the scheme of ^4^*I*_9/2_,^4^*I*_11/2_, and ^4^*F*_3/2_ Stark levels of Nd^3+^ ions, which occupy two positions with the site symmetry of *C*_2_ in the YScO_3_ crystal structure. Figure 5 shows the Stark energy for each of two basic Nd^3+^ optical centers with site symmetry of *C*_2_. The first center *C*_2_ (I) is formed in the result of substitution of Y^3+^ by Nd^3+^, and the second center *C*_2_ (II) is a result of the substitution of Sc^3+^ by Nd^3+^.

Both Nd^3+^ optical centers have a site symmetry of *C*_2_ but depend on Nd–O distance in a structural polyhedra where Nd^3+^ substitutes Y^3+^ (center I) or Sc^3+^ (center II). Isomorphic substitution of basic structural cations (Y^3+^ and Sc^3+^) by Nd^3+^ in a local position leads to changes in the crystal field around Nd^3+^ ions, but the type of local symmetry remains *C*_2_. So, we can distinguish two different optical centers *C*_2_ (I) and *C*_2_ (II) with almost equal lifetimes and values of Stark splitting of ^4^*I* _9/2_, ^4^*I* _11/2_, and ^4^*F*_3/2_ levels (Figure 4 and Figure 5).

### 3.2. Spectroscopy of Tm^3+^:YScO_3_

**Figure 6:**
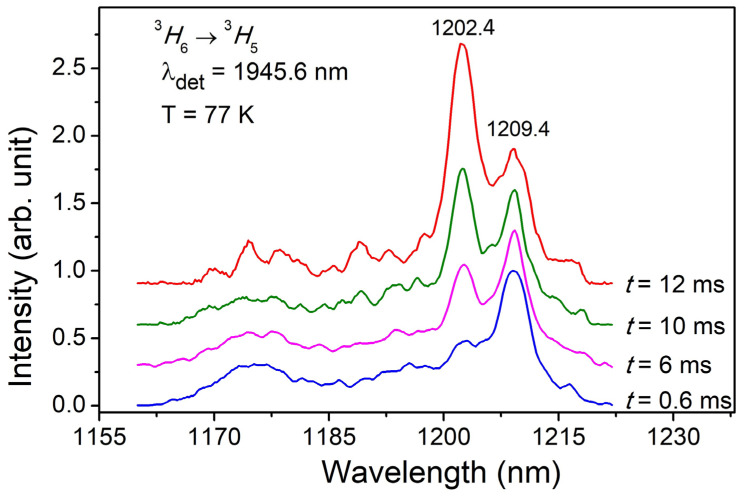
Normalized time-resolved luminescence excitation spectra of Tm^3+^:YScO_3_ being measured on the ^3^*H*_6_ *→* ^3^*H*_5_ transition at 77 K. Registration wavelength was 1945.6 nm. Delay times used were 0.6 ms, 6 ms, 10 ms and 12 ms.

Figure 6 shows low-temperature (T = 77 K) time-resolved luminescence excitation spectra of the 0.1 at. % Tm^3+^:YScO_3_ crystal fiber. The luminescence excitation spectra have been recorded at the LP-604 parametric laser radiation in the spectral range of 1160 to 1230 nm (^3^*H*_6_ *→* ^3^*H*_5_ transition) and registration of luminescence at 1945.6 nm (^3^*F*_4_ → ^3^*H*_6_ transition).

Changes in time delay of 0.6 ms to 12 ms relative to the moment of laser excitation lead to changes in luminescence excitation spectra, intensity redistribution between spectral lines of 1202.4 and 1209.4 nm. Changes being observed in luminescence excitation spectra allow us to assume that two types of optical centers of Tm^3+^ ions exist in the YScO_3_ crystal structure.

**Figure 7:**
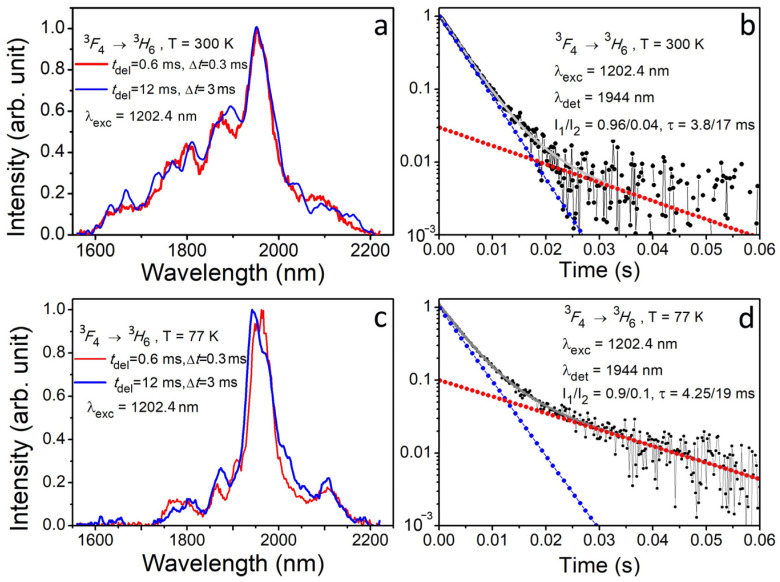
(**a**) Time-resolved luminescence spectra of Tm^3+^:YScO_3_ crystal fiber being measured on the ^3^*F*_4_ → ^3^*H*_6_ electron transition at excitation of 1202.4 nm (^3^*H*_6_ *→* ^3^*H*_5_ electron transition) and a temperature of 300 K. (**b**) Luminescence quenching kinetics of Tm^3+^:YScO_3_ being measured at excitation of 1202.4 nm and detection wavelengths of 1944 nm at 300 K. (**c**) Low-temperature (77 K) time-resolved luminescence spectra of Tm^3+^:YScO_3_ being measured at excitation of 1202.4 nm (^3^*H*_6_ *→* ^3^*H*_5_ electron transition). (**d**) Luminescence quenching kinetics of Tm^3+^:YScO_3_ being measured on the ^3^*F*_4_ → ^3^*H*_6_ electron transition at excitation of 1202.4 nm and detection wavelengths of 1944 nm at 77 K. For all spectra, a delay time and a gate used were *t*_del(1)_ = 0.6 ms and Δ*t*_(1)_ = 0.3 ms, respectively; *t*_del(2)_ = 12 ms and Δ*t*_(2)_ = 3 ms, respectively.

The luminescence of Tm^3+^:YScO_3_ has been measured on the ^3^*F*_4_ → ^3^*H*_6_ electron transition (spectral range of 1600–2240 nm) at selective laser excitation of 1202.4 and 1209.4 nm (^3^*H*_6_ *→* ^3^*H*_5_ electron transition). Figure 7a,c show time-resolved luminescence spectra of Tm^3+^:YSO_3_ being measured at temperatures of 77 and 300 K and laser excitation of 1202.35 nm. The luminescence spectra of Tm^3+^ ions show slight changes in spectral line profile. The luminescence line intensities are redistributing, depending on the time delay relative to the moment of laser excitation (0.6 to 12 ms). However, the Stark structure is poorly resolved. The low-temperature (77 K) luminescence spectra being measured on the ^3^*F*_4_ → ^3^*H*_6_ electron transition are non-structured (Figure 7c), as are the room-temperature (300 K) ones. To identify and assign the luminescence lines to specific optical centers has become a difficult task. First, the luminescence spectra of the Tm^3+^ ion include many Stark components of ^3^*F*_4_ (9 ones) and ^3^*H*_6_ (13 ones), and the mean values of Stark splitting are sufficiently low. Second, the spectral lines of the Tm^3+^ ion are broadened and overlapped due to high values of electron–phonon interaction.

Figure 7b,d show luminescence quenching kinetics of Tm^3+^:YScO_3_ being measured at excitation of 1202.4 nm (^3^*H*_6_ *→* ^3^*H*_5_ electron transition) and registration on 1944 nm in the ^3^*F*_4_ → ^3^*H*_6_ electron transition at temperatures of 300 and 77 K. Each luminescence quenching curve is described by two exponential models with lifetimes of τ (300 K) = 3.8/17 ms (*I*_1_/*I*_2_ = 0.96/0.04) and τ (77 K) = 4.25/19 ms (*I*_1_/*I*_2_ = 0.9/0.1). Short-time exponents (τ (300 K) = 3.8 and τ (77 K) = 4.25 ms) make a major contribution to luminescence quenching kinetics of the Tm^3+^ ion. Decreasing temperature down to 77 K leads to increasing the lifetime of the Tm^3+^ ion up to 11%. The contribution of the long-time component in the total intensity of luminescence quenching kinetics is low.

**Figure 8:**
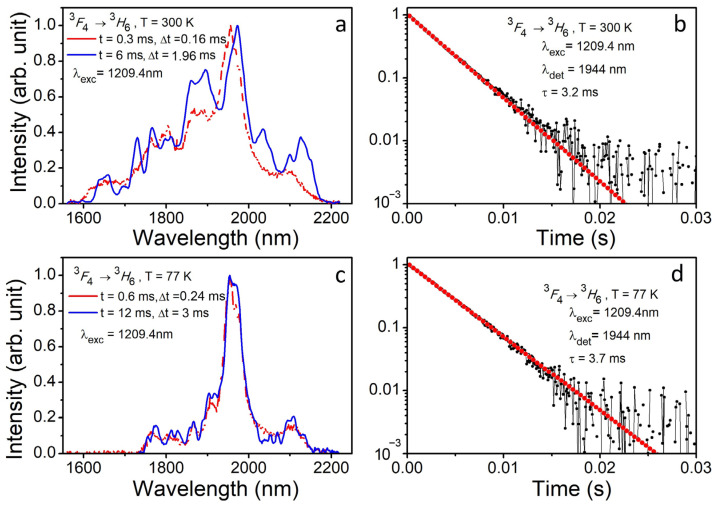
(**a**) Time-resolved luminescence spectra of Tm^3+^:YScO_3_ ion (^3^*F*_4_ → ^3^*H*_6_ electron transition) being measured at an excitation of 1209.4 nm (^3^*H*_6_ *→* ^3^*H*_5_ electron transition) at a temperature of 300 K. A delay time and a gate used were *t*_del(1)_ = 0.3 ms and Δ*t*_(1)_ = 0.16 ms, respectively; *t*_del(2)_ = 6 ms and Δ*t*_(2)_ = 1.96 ms, respectively. (**b**) Luminescence quenching kinetics of Tm^3+^ ion being measured at an excitation of 1209.4 nm and detection wavelengths of 1944 nm. (**c**) Time-resolved luminescence spectra of Tm^3+^:YScO_3_ being measured on the ^3^*H*_6_ *→* ^3^*H*_5_ electron transition at an excitation of 1209.4 nm and a temperature of 77 K. A delay time and a gate used were *t*_del(1)_ = 0.6 ms and Δ*t*_(1)_ = 0.24 ms, respectively; *t*_del(2)_ = 12 ms and Δ*t*_(2)_ = 3 ms, respectively. (**d**) Luminescence quenching kinetics of Tm^3+^:YScO_3_ being measured on the ^3^*F*_4_ → ^3^*H*_6_ electron transition at an excitation of 1209.4 nm and a detection of 1944 nm at 77 K.

Figure 8a,c show normalized time-resolved luminescence spectra of Tm^3+^:YScO_3_ being measured at excitation of 1209.4 nm (^3^*H*_6_ *→* ^3^*H*_5_ electron transition) and temperatures of 77 and 300 K. The luminescence spectra are modified, and the line intensities are redistributed with time delay. Decreasing the temperature down to 77 K does not improve structurization of luminescence spectra in the ^3^*F*_4_ → ^3^*H*_6_ electron transition in the Tm^3+^ ion (Figure 8c). Due to the poorly resolved Stark structure, identifying and assigning lines to specific centers is difficult. This is the result of the high value of electron–phonon interaction, overlapping spectral lines, and low values of splitting of ^3^*F*_4_ and ^3^*H*_6_ Stark levels.

Figure 8b,d show luminescence quenching kinetics in Tm^3+^:YScO_3_ at excitation of 1209.4 nm (^3^*H*_6_ *→* ^3^*H*_5_ electron transition) and registration of luminescence at 1944 nm (^3^*F*_4_ → ^3^*H*_6_ electron transition) at temperatures of 77 and 300 K. The luminescence quenching curves are described by a single exponent law with lifetimes of τ (300 K) = 3.2 and τ (77 K) = 3.7 ms at temperatures of 300 and 77 K, respectively. The difference in lifetime can be explained by the decrease in the intercenteral relaxation with temperature (down to 77 K).

Analysis of luminescence quenching kinetics of Tm^3+^:YScO_3_ (Figure 7b,d and Figure 8b,d) shows three types of Tm^3+^ optical centers in the crystal structure. Two of them are basic with short lifetimes of τ_1_ (300 K) = 3.2 ms, τ_1_ (77 K) = 3.7 ms and τ_2_ (300 K) = 3.8 ms, τ_2_ (77 K) = 4.25 ms. These centers belong to the low local symmetry of *C*_2_. One of them is formed as the result of the local substitution of Y^3+^ by Tm^3+^ and another one is due to the local substitution of Sc^3+^ by Tm^3+^. The third one has a local site of *C*_3*i*_ and lifetimes of τ_3_ (300 K) = 17 ms (*I* = 0.04) and τ_3_ (77 K) = 19 ms (*I* = 0.1) at 300 and 77 K, respectively. The third center has longer lifetime and higher local symmetry (*C*_3*i*_).

## 4. Conclusions

The substitution of Y^3+^ and Sc^3+^ by Nd^3+^ ions in the crystal structure of the YScO_3_ crystal fiber leads to the formation of three types of Nd^3+^ optical centers. Two of these dominate and exhibit a local symmetry of *C*_2_. The lifetimes of the Nd^3+^ optical centers in the YScO_3_ crystal fiber were determined at two different temperatures. For the center I, the lifetimes were measured to be τ_1_(300 K) = 250 μs and τ_1_(77 K) = 290 μs. For the center II, the lifetimes were determined to be τ_2_(300 K) = 240 μs and τ_2_(77 K) = 250 μs. In addition to the two dominant optical centers, a third optical center with shorter lifetimes has been observed in the YScO3 crystal fiber. The third center, likely a Nd^3+^–Nd^3+^ aggregate pair, exhibits lifetimes of *τ*_3_(300 K) = 100 μs and τ_3_(77 K) = 130 μs. This aggregate pair is believed to form as a result of the local substitution of Nd^3+^ for the basic structural cation (Y^3+^ and Sc^3+^) in the neighboring octahedra that share an edge. The quenching of Nd^3+^ luminescence is primarily ascribed to a combination of interaction between neighboring Nd^3+^ ions and the process of nonradiative relaxation of the excitation energy. The Stark component scheme for ^4^*F*_3/2_, ^4^*I*_11/2_, and ^4^*I*_9/2_ electron levels in the Nd^3+^ ion has been built for both basic optical centers with the local symmetry of *C*_2_.

Two short-lifetime optical centers of Tm^3+^ ions with the local symmetry of *C*_2_ are formed in the YScO_3_ crystal fiber through the substitution of Y^3+^ and Sc^3+^ with Tm^3+^ ions. The lifetimes of these optical centers have been estimated as τ_1_ (300 K) = 3.8 ms and τ_1_(77 K) = 4.25 ms for center I, and τ_2_ (300 K) = 3.2 ms and τ_2_ (77 K) = 3.7 ms for center II. The third center with long lifetimes of *τ*_3_ (300 K) = 17 ms and *τ*_3_ (77 K) = 19 ms has a local symmetry classified as *C*_3*i*_.

## Data Availability

Data sharing is not applicable for this article.
